# Multi-scale mathematical modelling of tumour growth and microenvironments in anti-angiogenic therapy

**DOI:** 10.1186/s12938-016-0275-x

**Published:** 2016-12-28

**Authors:** Yan Cai, Jie Zhang, Zhiyong Li

**Affiliations:** 1 State Key Laboratory of Bioelectronics, Southeast University, Nanjing, China; 2 School of Biological Sciences and Medical Engineering, Southeast University, 2 Sipailou, Nanjing, 200018 China; 3 Shanghai Pulmonary Hospital, Tongji University, Shanghai, China

**Keywords:** Multi-scale mathematical model, Numerical simulation, Solid tumour growth and angiogenesis, Tumour microenvironment, Anti-angiogenic therapy

## Abstract

**Background:**

Angiogenesis, a process of generation of new blood vessels from the pre-existing vasculature, has been demonstrated to be a basic prerequisite for sustainable growth and proliferation of tumour. Anti-angiogenic treatments show normalization of tumour vasculature and microenvironment at least transiently in both preclinical and clinical settings.

**Methods:**

In this study, we proposed a multi-scale mathematical model to simulate the dynamic changes of tumour microvasculature and microenvironment in response to anti-angiogenic drug endostatin (ES). We incorporated tumour growth, angiogenesis and vessel remodelling at tissue level, by coupling tumour cell phenotypes and endothelial cell behaviour in response to local chemical and haemodynamical microenvironment.

**Results:**

Computational simulation results showed the tumour morphology and growth curves in general tumour progression and following different anti-angiogenic drug strategies. Furthermore, different anti-angiogenic drug strategies were designed to test the influence of ES on tumour growth and morphology. The largest reduction of tumour size was found when ES is injected at simulation time 100, which was concomitant with the emergence of angiogenesis phase.

**Conclusion:**

The proposed model not only can predict detailed information of chemicals distribution and vessel remodelling, but also has the potential to specific anti-angiogenic drugs by modifying certain functional modules.

## Background

Angiogenesis is a physiological process of generation of new blood vessels required for normal body functioning, such as wound healing, endometrial growth during the menstrual cycle, tissue grafting, inflammation, and hypoxia. However, clinically and pathologically it has been demonstrated as a basic prerequisite for sustainable growth, proliferation and metastatic spread of solid tumours [1]. Cascade of events activated by several pro-angiogenic factors produced by hypoxic tumour cells happens during tumour angiogenesis including dissolution of vascular basal membrane, increased vascular permeability and degradation of extracellular matrix (ECM) resulting in endothelial cell (EC) migration, invasion, proliferation and tube formation [2]. The growth of new blood vessels requires the concerted action of activators and inhibitors of angiogenesis. Chemicals that can activate angiogenesis include vascular endothelial growth factor (VEGF), matrix metalloproteinases (MMPs), placenta growth factor (PlGF), fibroblast growth factor (FGF) and hepatocyte growth factor (HGF) [3]. Endogenous inhibitors of angiogenesis include angiostatin (AS) and endostatin (ES) [4]. The imbalance between angiogenic growth factors and inhibitors is believed to be significantly important in the development of the tumour vasculature [5].

Given the prominent role of angiogenesis in cancer, many cancer therapies aim to block angiogenesis and thereby inhibit tumour growth. These approaches undertaken towards that goal include inhibition of growth factors required for ECs proliferation and survival, such as VEGF [6]; inhibition of MMPs [7]; or direct inhibition of EC migration [8]. In addition, vascular disrupting agents (VDA) have been shown to inhibit tumour growth through induction of vascular collapse [9]. This research was transformed into therapeutic modality which led to the development of bevacizumab (Avastin), the first VEGF targeted anti-angiogenesis cancer therapy, and approved by the FDA in 2004 [10].

While the anti-angiogenic therapy have been approved for cancer treatment, it appears that the clinical application of anti-angiogenic therapy is more complex than originally thought. Since the anti-angiogenic drugs target tumour vasculatures without tumour cells, resistance to anti-angiogenic therapy caused by the pathological microenvironment in tumour is a prominent issue which may explains the variable results observed in the experiment and clinic using this approach [11]. Unlike normal blood vessels, tumour vasculature has abnormal structure and function [12]. The microvessels inside the tumour are leaky, which causes the high interstitial fluid pressure (IFP) in tumours. The blood perfusion in the abnormal microvasculature is heterogeneous and often compromised [13]. In addition, host-tumour interactions regulate the dynamic balance of angiogenic growth factors and inhibitors and result in the pathophysiological characteristics of the tumour [14]. Therefore, it is important to understand the mechanisms of interactions between tumour cells and microenvironment in response to anti-angiogenic therapy.

Mathematical modelling and numerical simulation offer powerful tools with which to study complex biological processes, such as tumour growth and angiogenesis. Billy et al. [15] developed a model of tumour growth that includes inhibitors (ES and Ang2) and promoters (VEGF and Ang1) of angiogenesis. Sleeman and coworkers’ [16] recent study proposed a model that combined angiogenesis and haemodynamic simulations in metastatic tumours. Their model predicted that treatment with angiostatin affected tumour vessels through the process called ‘vessel normalization’. Jain and coworkers developed a model to predict changes in tumour microenvironment including interstitial fluid pressure after vessel normalization treatment [17, 18]. Alarcon et al. [19] proposed a model of VEGFR association with VEGF and internalization to investigate how these processes influence the response to anti-angiogenic therapy. Using numerical algorithms, such as partial differential equations, agent-based modelling, and continuous–discrete hybrid modelling, computational modelling and numerical simulation provide platforms to study the complexity of tumour growth and angiogenesis process and the therapeutic strategies of anti-angiogenesis in cancer.

Based on the continuous–discrete hybrid model proposed by Chaplain’s group for modelling angiogenesis [20], we started our work by investigating the dynamic interactions between local haemodynamics and angiogenesis in a 2D model [21] and then developed it to a 3D model [22]. The inclusion of different tumour cell phenotypes into the angiogenesis simulation forms our second generation of model in which the tumour growth, vessel remodelling and blood perfusion were incorporated [23]. The present study is a further development in which a multi-scale 3D model was established aiming to simulate the dynamic changes of tumour microvasculature and microenvironment in response to anti-angiogenic therapy. The model focused on the anti-angiogenic drug endostatin (ES), which can inhibit endothelial cell (EC) proliferation, migration, invasion and tube formation. We envision that the proposed model will serve as a simulation framework for studying tumour growth and the changes of tumour microenvironment in response to anti-angiogenic therapy.

## Mathematical model and methods

**Fig. 1:**
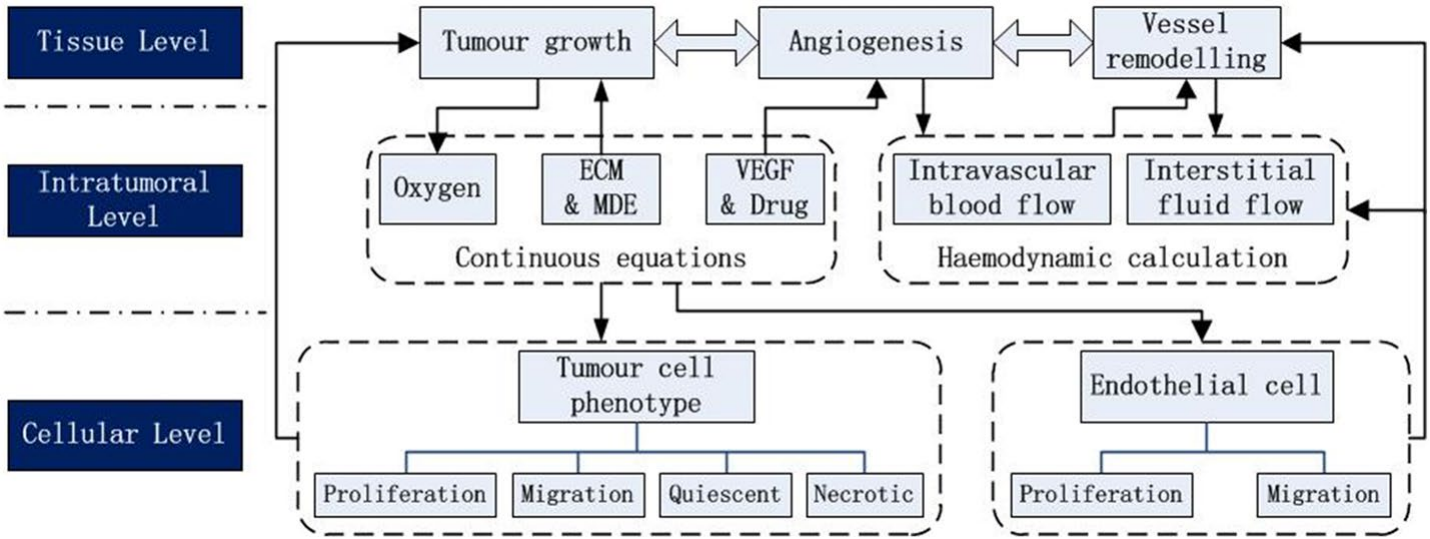
Structure of multi-scale modelling system

Multi-scale 3D tumour modelling is utilized to detail the mechanisms of tumour growth and angiogenesis. We have organized our model into three levels: tissue, intratumoral and cellular layers. The overall structure of this model is summarized in Fig. 1. In the tissue layer, we deal with the morphology of solid tumour and the structural properties of the vascular network. Four different phenotypes of tumour cells with corresponding metabolic characteristics are introduced in “Tumour growth” section. Modelling of tumour angiogenesis due to endothelial cell proliferation and migration is presented in “Angiogenesis and vessel remodelling” section. In addition, vessel remodelling in response to varied microenvironment is also considered in “Angiogenesis and vessel remodelling” section. The tissue level and the cellular level are coupled by the changes of microenvironment in the intratumoral level, including chemicals concentration flied (“Chemical microenvironment” section) and haemodynamic information (“Haemodynamical microenvironment” section). In “Anti-angiogenic therapy” section, we will develop the multi-scale model with anti-angiogenic drug delivery to investigate the influence of anti-angiogenic therapy on the tumour growth. Initial conditions and simulation algorithm are given in “Simulation setup” section.

### Tumour growth

The probabilistic hybrid model for tumour cell growth is based on the previous work [23]. We assumed four different phenotypes of tumour cells: the proliferative cells (PC), the quiescent cells (QC), the necrotic cells (NC) and the migrating cells (MC). Two thresholds of oxygen concentration for cell proliferation (θ_prol_) and cell survival (θ_surv_) are introduced to describe the effects of oxygen field on the tumour cell actions. Each phenotype of tumour cell has a different coefficient of oxygen consumption rate and the production rate of VEGF and MDEs (Table 1) [24].

**Table 1:** Parameters of different phenotypes of tumour cells

Phenotypes	MDE production	VEGF production	Oxygen consumption
Migrating cells (M)	2*μ*_T_	*χ* × 4	2*γ*
Proliferating cells (P)	*μ*_T_	*χ*	*γ*
Quiescent cells (Q)	*μ*_T_/5	*χ* × 2	*γ*/2
Necrotic cells (N)	*μ*_T_/10	*χ* × 4	*γ*/4

#### Proliferative cells (PC)

If there is enough oxygen ($C_{o}^{ex} \ge \uptheta_{\text{prol}}$) and space is available, a tumour cell will proliferate into two daughter cells with a probability, defined as T_age_/T_TC_. T_age_ is the tumour cell age, ranging from 1 to T_TC_ and with an incremental 1 in each simulation time step. T_TC_ is the tumour cell proliferation time (set to be 9 h, equal to six time steps). One of the two daughter cells will replace the parent cell and the other cell will move to a neighbouring element.

#### Quiescent cells (QC)

When a tumour cell satisfies the survival condition but there is no neighbouring space for it to proliferate, it will go quiescent. When the neighbouring space of one quiescent cell has been released, the quiescent cell will turn back into a proliferating cell if the local oxygen supply is sufficient.

#### Necrotic cells (NC)

When the local oxygen concentration at a tumour site is less than the cell survival threshold θ_surv_, the tumour cell is marked as a necrotic cell and will not be revisited at the next time step. A necrotic cell has a probability of 20% to disappear and release the space for a tumour cell or an endothelial cell if it stays necrotic for more than 45 h (30 time steps).

#### Migrating cells (MC)

When local oxygen level is higher than θ_surv_ but lower than θ_prol_ and a space is available, a proliferating cell has a probability (50%) to become a migrating cell (MC), and will migrate to a neighbouring space which has the highest oxygen concentration in the neighbouring elements. It was also assumed that the migrating cells adjacent to the pre-existing vessel wall have higher probability of moving in the longitudinal direction (vessel axial direction) than the radial direction. The migration speeds of the two directions are the same. After a migrating cell completes its movement, the space it originally occupied will be released for other cells.

### Angiogenesis and vessel remodelling

A 3D hybrid discrete–continuum angiogenesis model was adopted to investigate the EC migration and proliferation through random motility, chemotaxis in response to VEGF distributions and haptotaxis in response to the local ECM density [20]. Endothelial sprouting is only allowed in immature vessels, and the endothelial cell distribution was updated based on the equation

1 $$\frac{{\partial {\text{e}}}}{{\partial {\text{t}}}} = {\text{D}}_{\text{e}} \nabla^{2} {\text{e}} - \nabla \cdot \left( {\frac{{\upphi_{\text{c}} }}{{1 + \sigma {\text{C}}_{\text{v}} }}{\text{e}}\nabla {\text{C}}_{\text{v}} + \upphi_{\text{h}} {\text{e}}\nabla {\text{C}}_{\text{f}} } \right)$$

where e is the EC density. D_e_, $\upphi_{\text{c}}$, $\upphi_{\text{h}}$ are EC diffusion, chemotaxis and haptotaxis coefficients, respectively. ECs are allowed to move along the six directions in a 3D space.

In our model, we consider vessel dilation as the first sign of a pre-existing vessel becoming an immature vessel. A vessel segment inside the tumour has a VEGF concentration larger than a threshold θ_VEGF_ will increase its radius R with the rate of 0.4 µm h^−1^ which will stop when the vessel radius reaches the maximum value of R_max_ = 10 µm [25]. At the same time, the permeability of the vessel wall L_p_ is increasing in a dilation vessel, and satisfies

2 $${\text{L}}_{\text{p}} = \left\{ {\begin{array}{l} {{\text{L}}_{\text{P}}^{\text{T}} \left( {1 - \frac{{{\text{R}}_{ \hbox{max} } - {\text{R}}}}{{{\text{R}}_{ \text{max} } }}} \right), \quad {\text{immature }}\;{\text{vessel}}} \\ {{\text{L}}_{\text{P}}^{\text{N}} , \quad \quad \qquad \qquad \quad {\text{mature }}\;{\text{vessel}}} \\ \end{array} } \right.$$

where ${\text{L}}_{\text{p}}^{\text{N}}$ is the initial value of L_p_ referred to the vessel permeability value in the normal tissue; ${\text{L}}_{\text{p}}^{\text{T}}$ is the maximum value of L_p_ according to the experiments of vessel permeability value in a tumour microvessel.

For a pre-existing vessel, once vessel dilation occurs, the vessel segment is treated as an immature vessel with increased L_p_. In the simulation, vessel wall compliance is defined by the radius changing under the influence of intravascular and interstitial pressures and collapse pressure based on the empirical equation of Netti et al. [26].

3 $${\text{R}} = \left\{ {\begin{array}{l} {{\text{R}}_{0} \left( {\frac{{{\text{P}}_{{\text{v}}} - {\text{P}}_{{\text{i}}} + {\text{P}}_{{\text{c}}} }}{{\text{E}}}} \right)^{{\text{b}}} , \quad {\text{immature~vessel}}} \\ {{\text{R}}_{0} , \quad \quad \quad \quad \quad {\text{~mature~vessel}}} \\ \end{array} } \right.$$

where R_0_ is the origin radius of the capillary; P_c_ is the collapse pressure; b is the compliance exponent; E is the compliance coefficient. The intravascular and interstitial pressures are calculated by fully coupled haemodynamic simulation, which will be detailed in “Haemodynamical microenvironment” section.

Based on the above equations, when the vessel segment becomes immature, L_p_ will increase which causes higher P_i_, and consequently vessel will be compressed. A compressed vessel, on the other hand will induce a higher flow resistance, lower flow which will then decrease the wall shear stress (WSS) level for the vessel. Vessel collapse will occur by either a significant reduced R or WSS criteria [24].

### Chemical microenvironment

The tumour cell and endothelial cell behaviours are coupled by the changes of the chemicals in the extra-cellular matrix (ECM), such as oxygen, VEGF and matrix degradation enzymes (MDEs) [24]. The transport of these chemicals (oxygen, VEGF and MDEs) are modelled by quasi-steady reaction–diffusion equations. The ECM is treated as a continuous substance and can be degraded by MDEs, while the MDEs are governed by diffusion, produced by TCs and ECs, and the decay of itself. The equations describing the interactions of TCs and ECs with ECM, MDE and VEGF are

4 $$\frac{{\partial {\text{C}}_{\text{f}} }}{{\partial {\text{t}}}} = - \updelta {\text{C}}_{\text{m}} {\text{C}}_{\text{f}}$$

5 $$\frac{{\partial {\text{C}}_{\text{m}} }}{{\partial {\text{t}}}} = {\text{D}}_{\text{m}} \nabla^{2} {\text{C}}_{\text{m}} + \upmu_{\text{T}} {\text{TC}}_{{{\text{i}},{\text{j}}}} + \upmu_{\text{E}} {\text{EC}}_{{{\text{i}},{\text{j}}}} - \uplambda_{\text{m}} {\text{C}}_{\text{m}}$$

6 $$\frac{{\partial {\text{C}}_{\text{v}} }}{{\partial {\text{t}}}} = {\text{D}}_{\text{v}} \nabla^{2} {\text{C}}_{\text{v}} + \upchi {\text{TC}}_{{{\text{i}},{\text{j}}}} + \upxi {\text{C}}_{\text{f}} - \upepsilon {\text{EC}}_{{{\text{i}},{\text{j}}}} - \uplambda_{\text{v}} {\text{C}}_{\text{v}}$$

where C_f_, C_m_, C_v_ are the ECM, MDE and VEGF concentration, separately. The TC_i,j_ and EC_i,j_ terms represent a tumour cell and an endothelial cell located at a node position (i, j). Their values are either 1 if a cell is present or 0 if it is not. D_m_ is the MDE diffusion coefficient. D_v_ is VEGF diffusion coefficient. δ, μ_T_, μ_E_, λ_m_, χ, ξ, ɛ, λ_v_ are positive constants.

To obtain a more realistic oxygen concentration field, the advection and diffusion of oxygen in the vessel network are introduced [27]. The computational space is separated into three domains to characterize three distinct physiological processes, which are (a) the oxygen advection equation inside the vessel, (b) the oxygen flux across the vessel wall and (c) the free oxygen diffusion in the tissue. The detailed equations and methodology of oxygen delivery can be found in Cai et al. [28].

### Haemodynamical microenvironment

The haemodynamic model in this study is based on our previous work on the coupled modelling of intravascular blood flow with interstitial fluid flow [21, 22]. Briefly, the basic equation for the intravascular blood flow is the flux concentration and incompressible flow at each node. Flow resistance is assumed to follow Poiseuille’s law in each vessel segment. The interstitial fluid flow is controlled by Darcy’s law. The intravascular and interstitial flow is coupled by the transvascular flow, which is described by Starling’s law. Blood viscosity is a function of vessel diameter, local haematocrit, and plasma viscosity [29]. In addition, vessel compliance and wall shear stress are correlated to vessel remodelling and vessel collapse (see “Angiogenesis and vessel remodelling” section).

The main equations for blood flow calculation are as follows:

7 $${\text{Q}}_{\text{v}} = \frac{{\uppi {\text{R}}^{4} \Delta {\text{P}}_{\text{v}} }}{{8\upmu \Delta {\text{l}}}}$$

8 $${\text{Q}}_{\text{t}} = 2\uppi {\text{R}} \cdot \Delta {\text{l}} \cdot {\text{L}}_{\text{p}} ({\text{P}}_{\text{v}} - {\text{P}}_{\text{i}} - \upsigma_{\text{T}} (\uppi_{\text{v}} - \uppi_{\text{i}} ))$$

9 $${\text{Q}} = {\text{Q}}_{\text{v}} - {\text{Q}}_{\text{t}}$$

where Q is the flow rate of each vessel segment, which has a value zero at each node of the vessel network due to the assumption of flux conservation and incompressible flow. Q_v_ is the vascular flow rate without fluid leakage; Q_t_ is the transvascular flow rate. $\Delta {\text{l }}$ and R are the mean length and radius of the vessel segment. P_v_ and P_i_ are the intravascular pressure and the interstitial pressure, respectively. Lp is the hydraulic permeability of the vessel wall. σ_T_ is the average osmotic reflection coefficient for plasma proteins; π_v_ and π_i_ are the colloid osmotic pressure of plasma and interstitial fluid, respectively.

The velocity of intravascular U_v_ and interstitial flow U_i_ satisfies

10 $${\text{U}}_{\text{v}} = {{\text{Q}}/ {\uppi {\text{R}}^{2} }}$$

11 $${\text{U}}_{\text{i}} = - {\text{K}}\nabla {\text{P}}_{\text{i}}$$

12 $$\nabla \cdot {\text{U}}_{\text{i}} = \frac{{{\text{L}}_{\text{p}} {\text{S}}}}{\text{V}}({\text{P}}_{\text{v}} - {\text{P}}_{\text{i}} - \upsigma_{\text{T}} (\uppi_{\text{v}} - \uppi_{\text{i}} ))$$

where K is the hydraulic conductivity coefficient of the interstitium; S/V is the surface area per unit volume for transport in the interstitium.

### Anti-angiogenic therapy

Endostatin (ES), identified in mice by O’Reilly et al. [30] in 1997, has been considered as one of the most potential anti-angiogenic drugs. It can inhibit endothelial cell proliferation, migration, invasion and tube formation. In the present model, EC apoptosis in response to Endostatin is introduced in the angiogenesis equation [Eq. (1)]

13 $$\frac{{\partial {\text{e}}}}{{\partial {\text{t}}}} = {\text{D}}_{\text{e}} \nabla^{2} {\text{e}} - \nabla \cdot \left( {\frac{{\upphi_{\text{c}} }}{{1 + \upsigma {\text{C}}_{\text{v}} }}{\text{e}}\nabla {\text{C}}_{\text{v}} + \upphi_{\text{h}} {\text{e}}\nabla {\text{C}}_{\text{f}} } \right) + \propto_{\text{r}} \left( {1 - \frac{\text{e}}{{{\text{e}}_{0} }}} \right){\text{e}}\left( {1 - \frac{{\upepsilon_{ \text{max} } {\text{C}}_{\text{ES}} }}{{{\text{C}}_{\text{ES}} {\text{C}}_{{{\text{ES}}50}} + {\text{C}}_{\text{ES}} }}} \right)$$

where e_0_ is the initial EC density. ɛ_max_ is the maximum inhibiting effect of ES on ECs. C_ES50_ is the ES concentration that induces 50% of the maximum inhibiting effect. C_ES_ is the concentration of ES, which satisfies

14 $$\frac{{\partial {\text{C}}_{\text{ES}} }}{{\partial {\text{t}}}} = \nabla \cdot \left( {{\text{D}}_{\text{ES}} \nabla {\text{C}}_{\text{ES}} } \right) + \frac{{ - {\text{R}}_{\text{ES}} {\text{C}}_{\text{ES}} + {\text{U}}_{{{\text{I}},{\text{ex}}}} }}{{{\text{V}}_{\text{p}} }} - \uplambda_{\text{ES}} {\text{C}}_{\text{ES}}$$

D_ES_ is the diffusion coefficient of ES. R_ES_ is the ES elimination rate in the plasma. U_I,ex_ is the ES injection rate. V_p_ is the volume of the plasma. λ_ES_ is the positive coefficient for ES decay of itself.

### Simulation setup

**Fig. 2:**
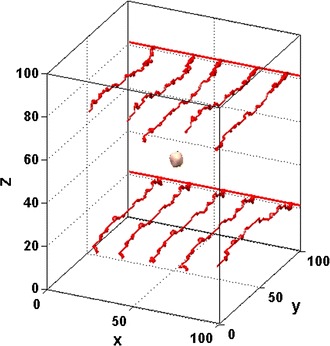
The initial pre-existing vasculature and tumour morphology of the model

The 3D model is defined on a 100 × 100 × 100 grid to cover a 1 mm × 1 mm × 1 mm volume, so the grid length corresponds approximately to the size of a tumour cell, i.e. 10 μm. Two separate lattices are constructed for tumour cells (TCs) and endothelial cells (ECs), with the constraint that each grid can contain only one single cell at each time point. We assume that there are two main parallel arterioles located at plane y = 100, which branch to form capillaries as initial pre-existing vasculature of the model (Fig. 2).

Dirichlet boundary conditions of the chemicals are used in the simulation field. The initial condition of ECM density is set to be 1and other chemicals’ concentration (oxygen, VEGF and MDEs) are 0. The chemicals’ concentrations are solved to steady state at each time step of the simulation with an inner iteration step of 5 s. The oxygen concentration has been normalized to be 0–1 in the "Results" section.

The total difference of P_v_ through the two main parallel arterioles from plane x = 100 to x = 0 is set to be 3.5 mmHg as the driving force of blood in the network (or the boundary condition). The distribution of red blood cells (RBCs) at a microvascular bifurcation is calculated based on the blood rheology simulation (see Wu et al. [21]).

Initially, 20 proliferating cells are placed in the central computational area (see the tumour morphology in Fig. 2). As the tumour grew with time, the metabolic microenvironment such as oxygen, VEGF and other chemicals concentrations are calculated accordingly. At the same time, tumour vessels undergo remodelling and collapse in response to both chemical environment and haemodynamical environment. As a consequence, the local oxygen supply changes with the updated tumour vasculature, which determines the different phenotypes of tumour cells in next time step. The initial concentration of anti-angiogenic drug (ES) is ${\text{e}}_{0} = 2.0 \times 10^{ - 9} {\text{mol}}\,{\text{L}}^{ - 1}$, and assumed of having an injection dose of ES* = 20 mg kg^−1^ day^−1^ after different time point of tumour growth (T_inj_ = 50, T_inj_ = 100 and T_inj_ = 150). All simulations were performed on an Intel Xeon 3.2 GHz CPU, 24 GB Memory desktop. The parameter values of baseline model are listed in Table 2.

**Table 2:** Parameter values used in the simulation

Parameter	Value	Description	Reference
Δl	10 μm	Lattice constant	
R_0_	4 μm	Origin radius of the capillary	
D_e_	10^−9^ cm^−3^ s^−1^	EC diffusion coefficient	[20]
$\upphi_{\text{c}}$	2.6 × 10^3^ cm^−3^ M^−1^ s^−1^	EC chemotaxis coefficient	[20]
$\upphi_{\text{h}}$	10^3^ cm^−3^ M^−1^ s^−1^	EC haptotaxis coefficient	[20]
${\text{L}}_{\text{p}}^{\text{T}}$	2.8 × 10^−7^ cm (mmHg s)^−1^	Vessel permeability in tumour tissue	[31]
${\text{L}}_{\text{p}}^{\text{N}}$	0.36 × 10^−7^ cm (mmHg s)^−1^	Vessel permeability in normal tissue	[31]
P_c_	3 mmHg	Vessel collapse pressure	[26]
E	6.5 mmHg	Vessel compliance coefficient	[26]
b	0.1	Vessel compliance index	[26]
D_m_	10^−9^ cm^−3^ s^−1^	MDE diffusion coefficient	[24]
δ	1.3 × 10^2^ cm^−3^ M^−1^ s^−1^	ECM degradation coefficient	[23]
μ_T_	1.7 × 10^−18^ Mcells^−1^ s^−1^	MDE production by TC	[23]
μ_E_	0.3 × 10^−18^ Mcells^−1^ s^−1^	MDE production by EC	[23]
λ	1.7 × 10^−8^ s^−1^	MDE decay coefficient	[24]
D_v_	2.9 × 10^−7^ cm^−3^ s^−1^	VEGF diffusion coefficient	[20]
χ	10^−17^ Mcells^−1^ s^−1^	VEGF production by TC	[32]
ξ	10^−3^ cm^−3^ s^−1^	VEGF production in ECM	[23]
ε	10^−20^ Mcells^−1^ s^−1^	VEGF consumption by EC	[32]
θ	10^−8^ s^−1^	VEGF decay coefficient	[32]
e_0_	2.0 × 10^−9^ mol L^−1^	Initial EC density	[33]
ɛ_max_	1	Max inhibiting effect of ES on ECs	[33]
C_ES50_	2.288 × 10^−8^ mol L^−1^	ES concentration that induces 50% of the maximum inhibiting effect	[33]
D_ES_	2.9 × 10^−7^ cm^−3^ s^−1^	Diffusion coefficient of ES	[33]
R_ES_	5.54 × 10^−5^ L s^−1^	ES elimination rate in the plasma	[33]
U_I,ex_	20 mg (kg × day)^−1^	ES injection rate	[33]
V_p_	10^−3^ L	Volume of the plasma	[33]
λ_ES_	10^−8^/s	ES decay coefficient	Estimated

## Results

### Tumour growth and angiogenesis without anti-angiogenic drug

**Fig. 3:**
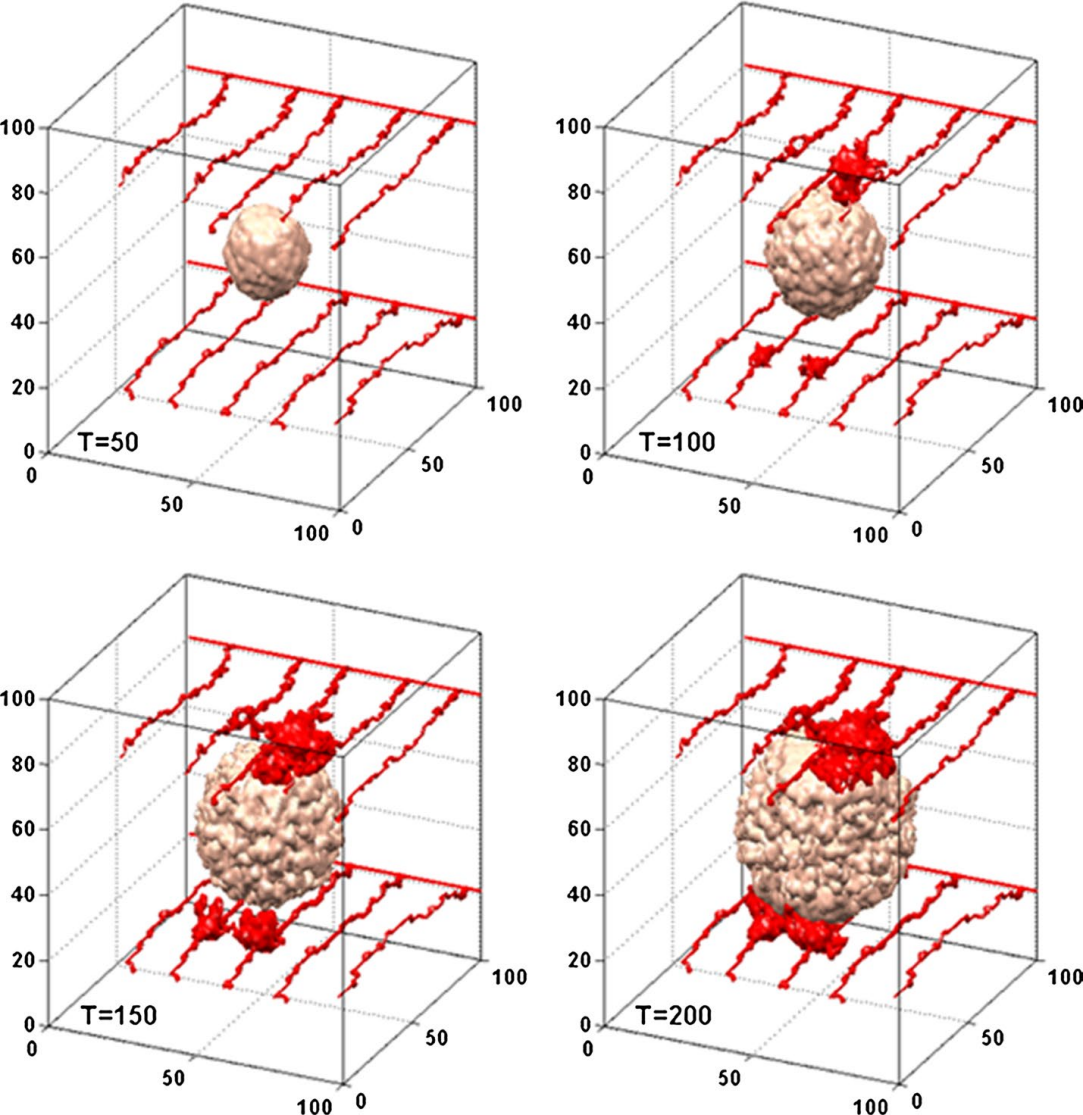
Time sequence of tumour morphology and angiogenesis during the simulation. Tumour vessels are represented by *red tubes*

Figure 3 shows the history of tumour growth and angiogenesis without anti-angiogenic drug. Tumour vessels are represented by red tubes. At the early stage, tumour cells rapidly uptake oxygen diffused from pre-existing vessels, which is known as avascular stage of tumour growth (T = 50). The angiogenesis phase is triggered on due to the drop of oxygen levels. Well circulated angiogenic vessel networks can be seen at T = 100, which suggests that the neo-vasculature generates a new supply of oxygen for further tumour growth. In addition, vascularized tumours are more likely to develop dendritic structures compared to round tumours in avascular phase. This indicates that tumour angiogenesis may reinforce the heterogeneity of tumour microenvironment, favoring a more aggressive morphology of solid tumours (T = 200).

**Fig. 4:**
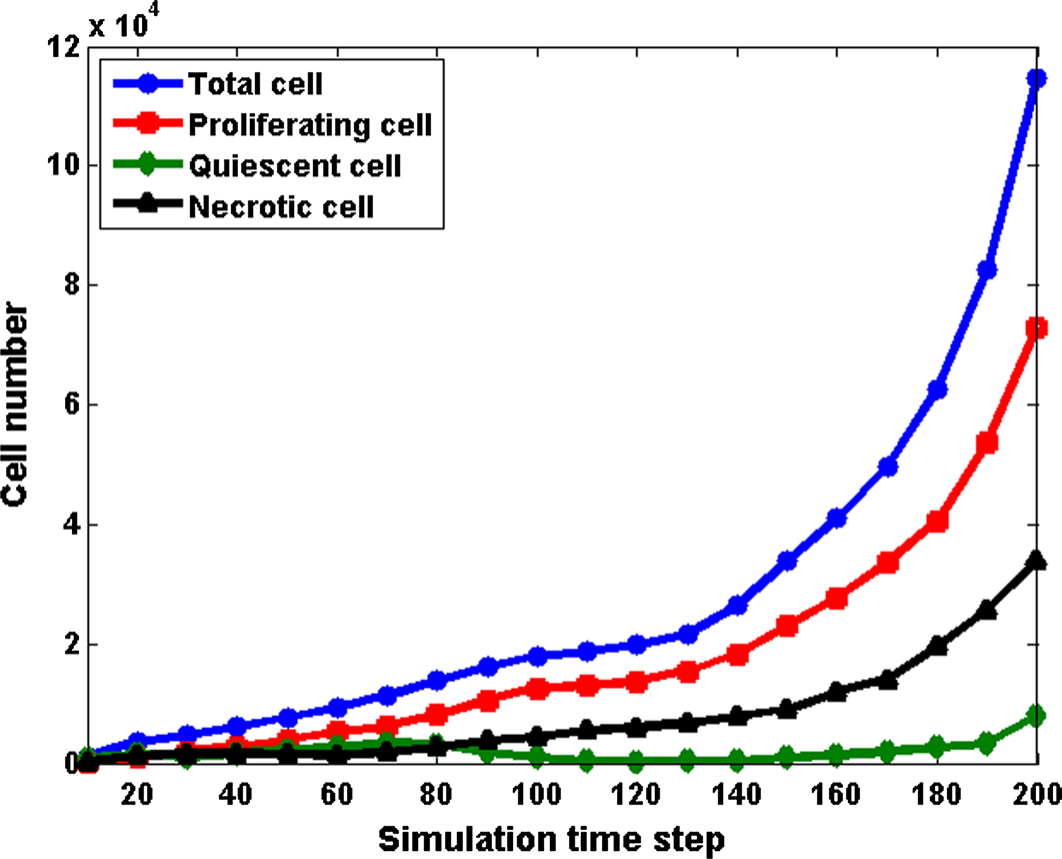
Growth history of tumour cells with different phenotypes. *Blue* total tumour cells; *red* proliferating cells; *green* quiescent cells; *black* necrotic cells

The numbers of tumour cells with different phenotypes at different time step are presented as curves in Fig. 4. The number of total tumour cells and proliferating cells increase continually with time in the simulation. However, the number of quiescent cells have a slight reduction in the period of T = 70 to T = 100. At the same time, necrotic cells remain linear expansion, due to the inadequate oxygen supply by pre-existing vessel network. As a consequence, angiogenesis phase occurs at this time (see Fig. 3). The proliferating cell population shows exponential increase after the emergence of angiogenic tumour vessels, which demonstrates that the feedback mechanisms of the coupled model work well in the simulation.

### Tumour growth and angiogenesis with anti-angiogenic drug

**Fig. 5:**
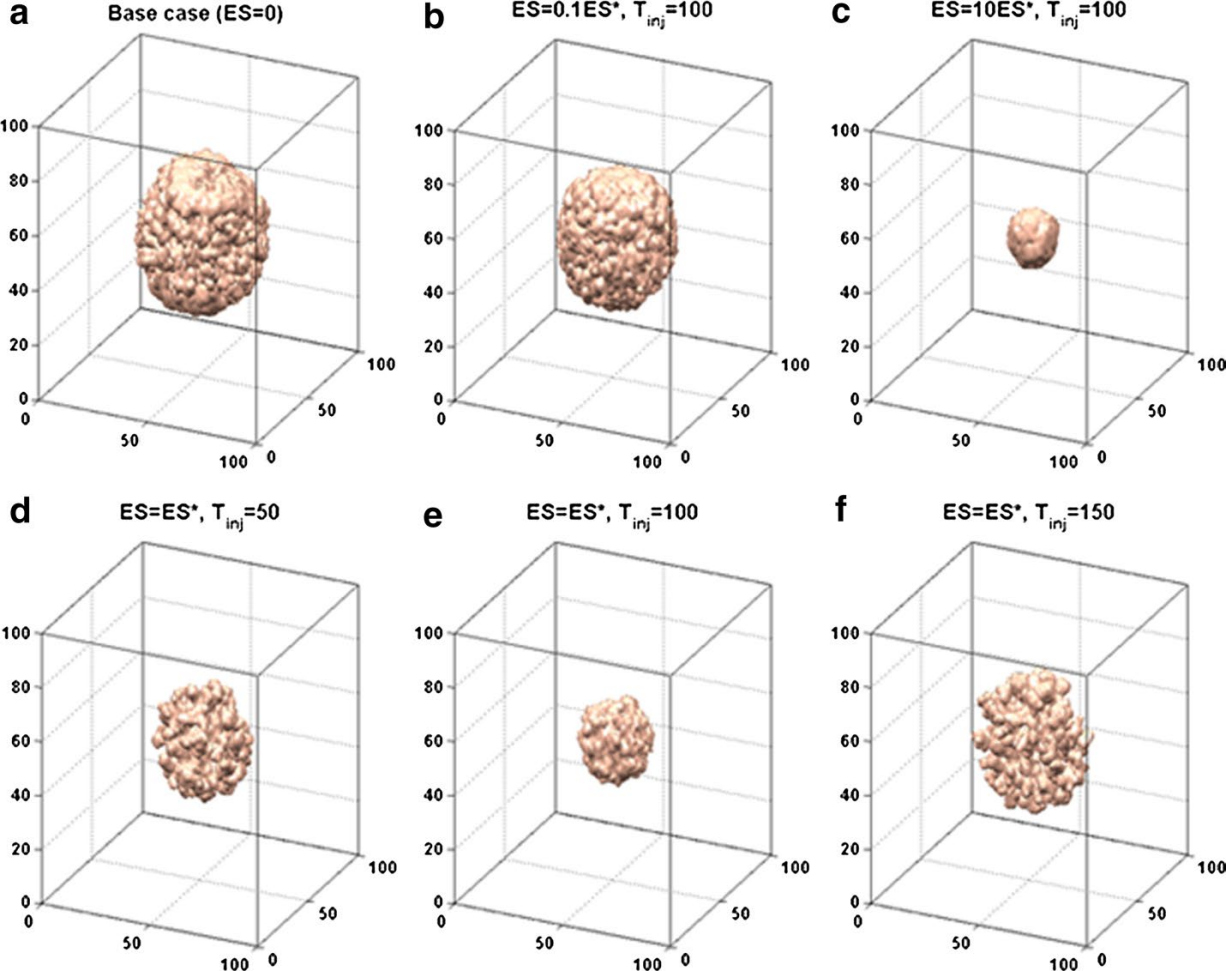
Tumour morphology at T = 200 following different anti-angiogenic drug strategies. **a** Base case with no anti-angiogenic drug, as described in “Tumour growth and angiogenesis without anti-angiogenic drug” section. **b**, **c** The anti-angiogenic drug ES is inserted at T_inj_ = 100, with different drug concentrations (ES = 0.1ES*, ES = 10ES*). **d**, **e**, **f** The effect of ES on tumour growth with same dose but different injection time, T_inj_ = 50, 100, 150 respectively

**Fig. 6:**
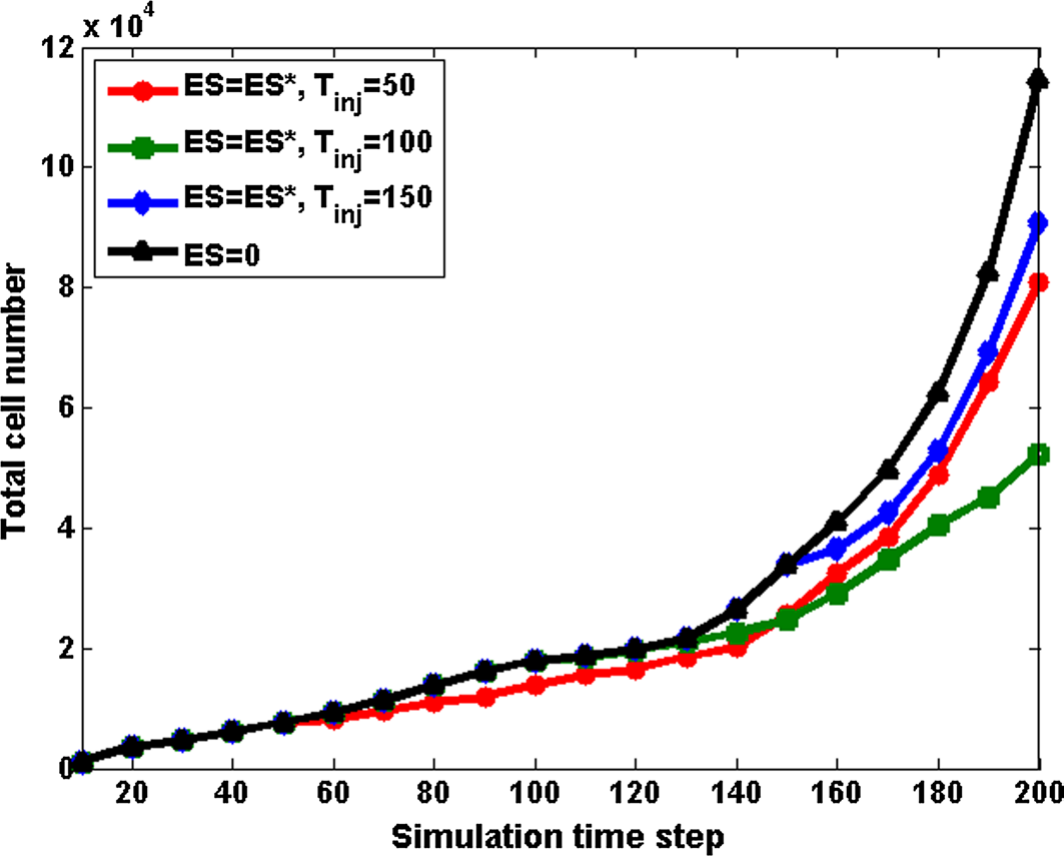
Growth history curves of total tumour cells following different anti-angiogenic drug strategies

In order to study the influence of anti-angiogenic drug ES on tumour growth and morphology, we tested five drug strategies with different drug concentration and different injection time. Compared to base case (Fig. 5a), a low ES concentration is found to induce little growth suppression (Fig. 5b), while large doses of ES (ES = 10ES*) can produce significant tumour cell apoptosis (Fig. 5c). The effect of different injection time of ES on tumour growth is shown in Fig. 5d–f. It is noteworthy that a drastically suppression of tumour size is generated when ES is inserted at simulation time 100, which is concomitant with the emergence of angiogenesis phase. As shown by growth history curves in Fig. 6, up to 50% of reduction of total tumour cells number can be seen after ES treatment on T_inj_ = 100. However, only 30 and 21% of reduction can achieved respectively if injection time of ES is earlier or later than simulation time 100.

### Metabolical and haemodynamical microenvironment

**Fig. 7:**
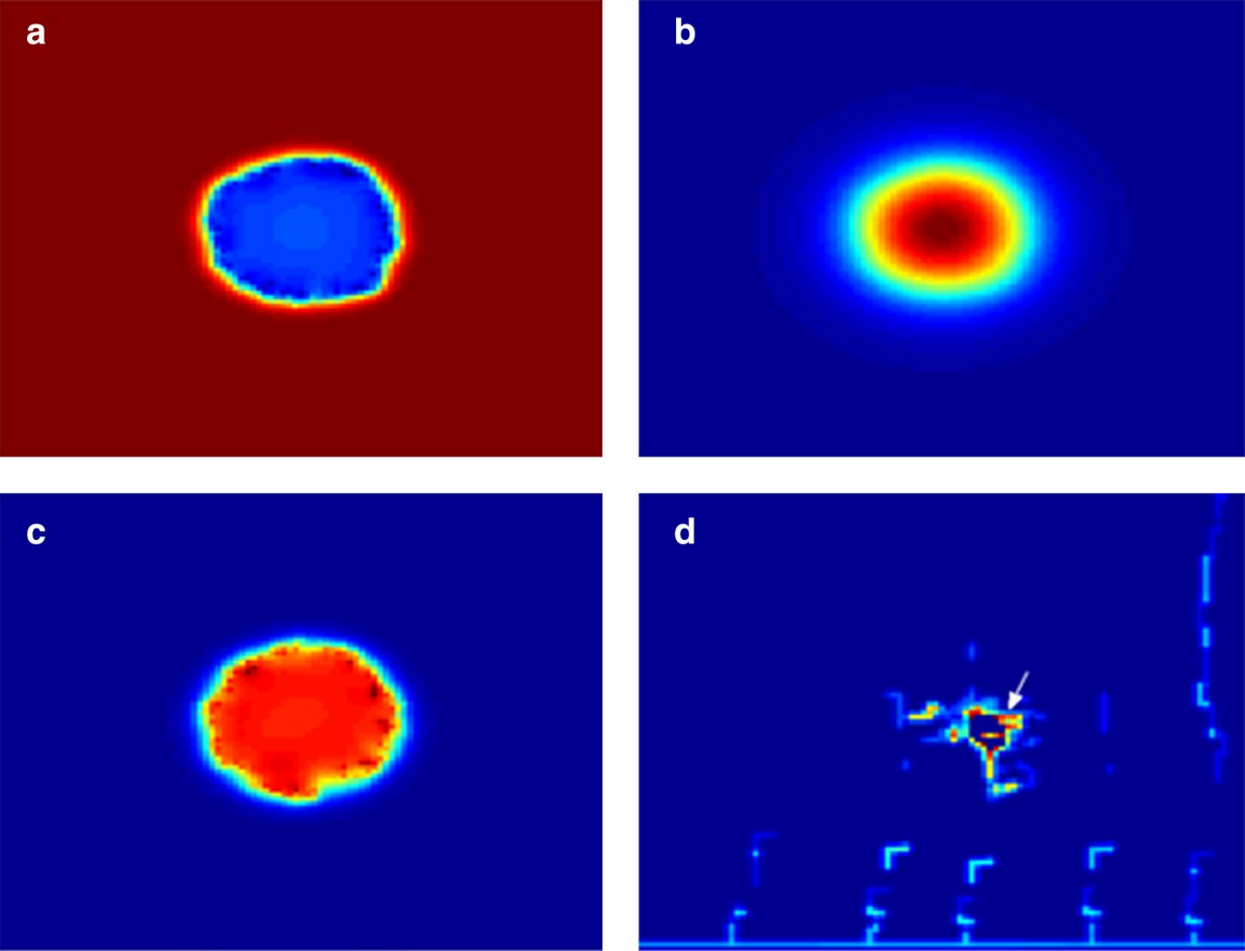
Chemical and haemodynamical microenvironment on T = 200, at plane z = 20. **a** Oxygen distribution. **b** VEGF distribution. **c** Interstitial pressure. **d** Vessel radius distribution. *Arrow* indicates the vessel dilation. Intensity: *red* (*high*), *blue* (*low*)

The model predicts the chemical and haemodynamical substances distribution including oxygen, VEGF, interstitial pressure and vessel radius, as shown in Fig. 7. The tumour centre is always in a hypoxic state, while high concentration of oxygen occurred near the tumour edge due to the localized angiogenic vasculature at the tumour periphery (Fig. 7a). VEGF has a high density inside the tumour associated with hypoxic tumour cells, attracting neo-vessels growth towards the tumour (Fig. 7b). Interstitial pressure P_i_ is significantly higher than that of surrounding tissue and drops dramatically in the tumour margin (Fig. 7c), which compromises the delivery of therapeutic agents both across the blood vessel wall and interstitium in tumours. Vessel radius shows unevenly distributed, with few dilated functional vessels left in the tumour centre (Fig. 7d, arrow). The large variation in vessel diameters will increase the flow resistance in tumours microvasculature, which has been observed in published experiments [5].

### Parameter sensitivity

**Fig. 8:**
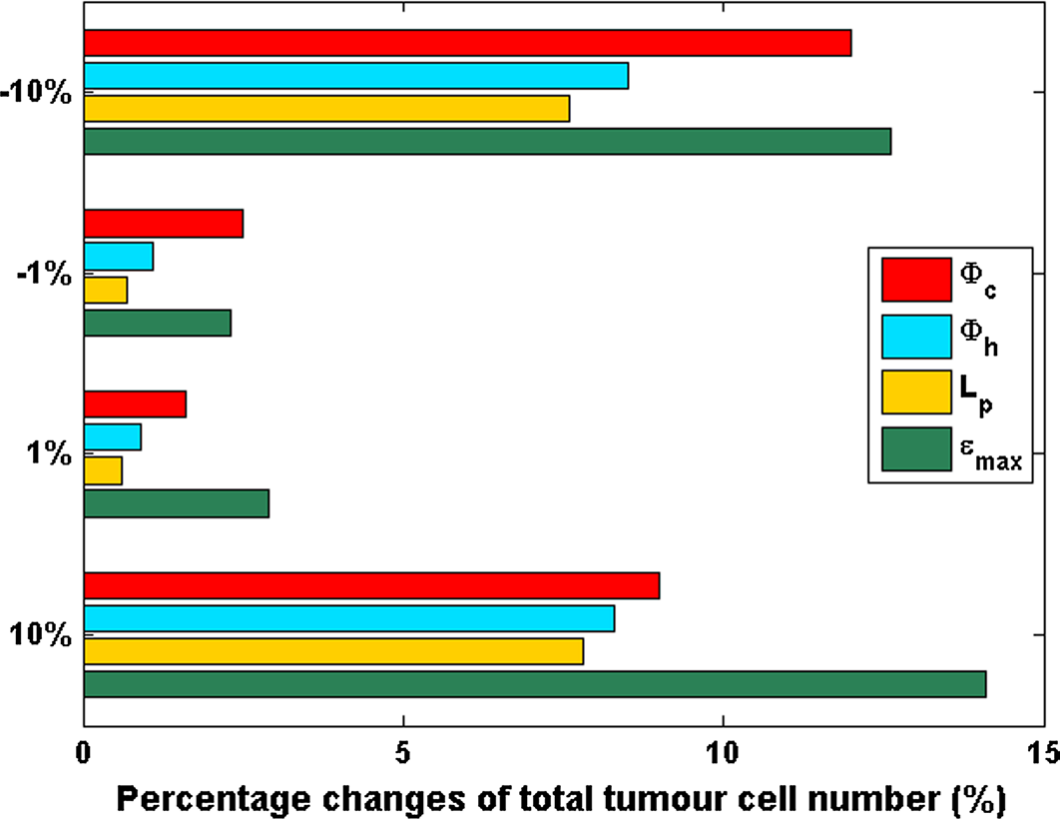
Percentage changes of number of total tumour cells with respect to varied parameters

In the current model, the main factors that directly influence ECs distribution are the chemotaxis of VEGF, the haptotaxis of ECM, the vessel remodelling in response to local haemodynamical microenvironment and the inhibition effect due to ES treatment. The sensitivity of tumour growth to the important parameters are analyzed, including chemotaxis coefficient $\upphi_{\text{c}}$, haptotaxis coefficient $\upphi_{\text{h}}$, wall permeability of tumour capillaries L_p_ and maximum inhibiting effect of ES ɛ_max_. The variation ranges of tested parameters are ±1 and ±10% from their default values listed in Table 2. As shown in Fig. 8, the number of total tumour cells is quite robust with respect to varied parameters (less than 15% changes). The most sensitive parameter to this model is the maximum inhibiting effect of ES ɛ_max_.

## Discussion

In this paper, we proposed a multi-scale coupled modelling system to investigate the dynamic process of tumour growth and angiogenesis in response to the changes in chemical and haemodynamical microenvironment caused by anti-angiogenic therapy. At tissue level, we considered tumour growth, tumour angiogenesis and vessel remodelling. Tumour growth and neo-vessel growth are coupled by the chemical microenvironment including oxygen, VEGF, ECM and MDE, which are described by continuous partial differential equations. At the same time, haemodynamic calculation is carried out by coupling intravascular blood flow and interstitial fluid flow. According to the microenvironmental information, detailed and comprehensive mechanisms of tumour cell phenotype and endothelial cell proliferation and migration are generated at cellular level, which in turn influence the modelling at intratumoral and tissue levels. In addition, we extended the model to study the dynamic changes of tumour growth in response to anti-angiogenic therapy. Anti-angiogenic drug ES is assumed to target immature vessels and may cause endothelial cell apoptosis.

Simulation results revealed multiple characteristics of tumour progression, including tumour expansion, morphology changes and angiogenesis. The growth curves of cell population with different cell phenotypes are consistent with the pathophysiological knowledge. Furthermore, different anti-angiogenic drug strategies are designed to test the influence of ES on tumour growth and morphology. The largest reduction of tumour size is found when ES is injected at simulation time 100, which is concomitant with the emergence of angiogenesis phase. With pharmacokinetics information of specific anti-angiogenic drugs, the proposed model can be adopted for cancer drug discovery and testing.

There are a few major limitations of the present work. On calculating the tumour mechanical environment, only interstitial fluid static pressure was included. Mechanical stress caused by the rapid proliferation of tumour cells was not simulated in the model. This solid mechanical stress may influence tumour cell behaviour. In addition, although most of the simulation parameters were set based on published experimental data, some of them cannot be found, such as tumour migration speed.

## Conclusions

A multi-scale coupled modelling system to investigate the dynamic process of tumour growth and angiogenesis in response to the changes in chemical and haemodynamical microenvironment during the anti-angiogenic therapy, is established. The simulation results demonstrate the inhibition of tumour growth and progression due to the reduction of neo-vasculature in response to anti-angiogenic therapy. Different anti-angiogenic drug strategies can be designed based on the proposed mathematical model, to test the influence of anti-angiogenic drug on tumour growth and morphology, with pharmacokinetics information of specific drugs
